# Symptomatic Carotid Atherosclerotic Plaques Are Associated With Increased Infiltration of Natural Killer (NK) Cells and Higher Serum Levels of NK Activating Receptor Ligands

**DOI:** 10.3389/fimmu.2019.01503

**Published:** 2019-07-12

**Authors:** Irene Bonaccorsi, Domenico Spinelli, Claudia Cantoni, Chiara Barillà, Narayana Pipitò, Claudia De Pasquale, Daniela Oliveri, Riccardo Cavaliere, Paolo Carrega, Filippo Benedetto, Guido Ferlazzo

**Affiliations:** ^1^Laboratory of Immunology and Biotherapy, Department Human Pathology, University of Messina, Messina, Italy; ^2^Research Center Cell Factory UniMe, University of Messina, Messina, Italy; ^3^Unit of Vascular Surgery, Department of Biomedical and Dental Sciences and Morphofunctional Imaging, University of Messina, Messina, Italy; ^4^Department of Experimental Medicine, Center of Excellence for Biomedical Research, University of Genoa, Genoa, Italy; ^5^IRCCS, Istituto Giannina Gaslini, Genoa, Italy; ^6^Clinical Pathology Unit, University Hospital – A.O.U. Policlinico G. Martino, Messina, Italy

**Keywords:** natural killer cells, atherosclerosis, carotid atherosclerotic plaques, natural killer cell activating receptors, interferon-γ, metalloproteases

## Abstract

A wide array of immune cells, including lymphocytes, is known to be present and to play a pathogenetic role in atherosclerotic lesions. However, limited information is currently available regarding the presence of Natural Killer (NK) cell subsets within vessel plaque, and more in general, regarding their role in human atherosclerosis. We evaluated the distribution of NK cells in human carotid atherosclerotic plaques, dissecting asymptomatic and symptomatic patients (identified as affected by stroke, transient ischemic attack, or amaurosis fugax within 6 months) with the aim of shedding light on the putative contribution of NK cells to the pathogenic process that leads to plaque instability and subsequent clinical complications. We observed that carotid plaques were consistently infiltrated by NK cells and, among them, CD56^bright^perforin^low^ NK cells were abundantly present and displayed different markers of tissue residency (i.e., CD103 CD69 and CD49a). Interestingly, carotid atherosclerotic plaques of symptomatic patients showed a higher content of NK cells and an increased ratio between CD56^bright^perforin^low^ NK cells and their CD56^dim^perforin^high^ counterpart. NK cells isolated from plaques of symptomatic patients were also stronger producers of IFN-γ. Analysis of the expression of NK activating receptor ligands (including MICA/B, ULBP-3, and B7-H6) in atherosclerotic carotid plaques revealed that they were abundantly expressed by a HLA-DR^+^CD11c^+^ myeloid cell population resident in the plaques. Remarkably, sera of symptomatic patients contained significant higher levels of soluble ligands for NK activating receptors. Our observations indicate that CD56^bright^ NK cells accumulate within human atherosclerotic lesions and suggest a possible contribution of NK cells to the process determining plaque instability.

## Introduction

Carotid atherosclerosisis is strongly associated with stroke and cerebrovascular disease. Nevertheless, some carotid atherosclerotic plaques (CAP) are stable and unlikely to produce symptoms, whereas others are unstable and may induce increased incidence of vascular complications. Symptomatic plaques generally reveal intima rupture, a thinner fibrous cap and a large necrotic core infiltrated by macrophages and lymphocytes. However, the complex molecular and cellular events underlying plaque destabilization are not completely elucidated and different immunological/inflammatory mechanisms are probably responsible for the clinical complications associated with unstable atherosclerotic plaques (AP). To further clarify the multiple causes of carotid plaque destabilization, it would be desirable to define a pathogenetic signature by characterizing phenotypic and functional features of cells taking part not only to plaque formation but also to the occurrence of patient's symptoms.

Immune cells, including innate lymphocytes such as Natural Killer (NK) cells, have been previously reported to infiltrate atherosclerotic lesions (1–3). NK cells are large granular lymphocytes, divided in two main subsets: CD56^dim^CD16^+^cells are the majority in human peripheral blood (PB) (≥95%) (4), while CD56^bright^CD16^−^ cells account for around 5% of NK cells in PB but represent the majority of NK cells in several peripheral tissues and in secondary lymphoid organs (5–7). CD56^dim^CD16^+^NK cells display potent cytolytic activity, while the CD56^bright^CD16^−^ counterpart is mainly responsible for cytokine production, primarily IFN-γ and TNF-α, but is poorly cytolytic (6, 8). Although NK cells were mainly described in PB and, more recently, in lymphoid tissues (8–10), it is now well-established that NK cells can be recruited at inflammatory sites (8, 10–12) and even enduringly reside in peripheral tissues at steady state (13–15).

The presence of NK cells in APs has been previously reported (1, 16–18) and it has also been shown that NKG2D, a potent immune-activating receptor mainly expressed by cytotoxic lymphocytes, including NK cells, plays a role in the vicious cycle of chronic inflammation that promotes atherosclerosis, since blocking NKG2D in mice reduced inflammation and plaque progression in atherosclerotic lesions of aorta (19).

It is noteworthy that Major Histocompatibility Complex (MHC) class I-related chains A and B (MICA/B), which represent major ligands of NKG2D receptor, are highly expressed in foam cells infiltrating the fibrous cap as well as in the intra-plaque hemorrhage space in atherosclerotic aortas of patients with type 2 diabetes mellitus (19). In agreement with these results, the expression of MICA/B in human phagocytes was also confirmed *in vitro* on foam cells derived from macrophages exposed to modified low density lipoproteins (20). All these findings suggest a role for NKG2D in enhancing the inflammatory state in atherosclerosis disease and support the hypothesis that NK cells might be actively engaged in atherosclerotic lesions.

Given that other ligands for activating receptors specifically expressed by NK cells, such as the NKp30 ligand B7-H6, have been shown to be present on macrophage surface under inflammatory conditions (21–23), we can hypothesize that induction of these ligands might even occur in the context of the inflammatory network of atherosclerosis.

Thus, on the basis of a possible contribution played by NK cells in the pathogenesis of atherosclerosis, the purpose of this study was to investigate whether the frequency and functions of NK cells infiltrating CAP might correlate with clinical complications occurring in the patients.

## Materials and Methods

### Patients and Samples

Fifty patients undergoing carotid endarterectomy at the Vascular Surgery Unit of the University Hospital *G. Martino* of Messina were enrolled in the study.

Patients were selected for invasive treatment according to the European Society for Vascular Surgery (ESVS) guidelines (24). Patients were evaluated with duplex-ultrasound. The risk of stroke was predicted estimating the diameter reduction of internal carotid artery (ICA) and evaluating plaque morphology (25). Patients with symptoms of stroke, transient ischemic attack (TIA) or amaurosis fugax within 6 months since diagnosis of carotid artery disease were defined symptomatic (26–28).

All patients were admitted in the Vascular Surgery ward of the University Hospital *G. Martino* of Messina 1 day before the intervention. Carotid plaques were removed by eversion carotid endarterectomy technique (24, 29) and pre-operative blood samples were obtained from all patients. As control, PB samples were obtained from age-matched individuals with a similar gender distribution and <40% of carotid stenosis assessed by ultrasonographic study. The study was approved by our Institutional Ethics Committee and all patients gave their written informed consent according to the Declaration of Helsinki.

### Carotid Plaque and Blood Sample Processing

After surgical removal, carotid plaques were extensively washed in phosphate-buffered saline (PBS) to remove cell debris and red blood cells (RBC) aggregates. Samples were then mechanically minced by scissors to obtain small fragments. In order to minimize blood contamination, tissue specimens were extensively rinsed after initial tissue slashing in small fragments. Then, samples were enzymatically digested with a mixture containing DNAse (100 μg/ml; Roche Diagnostics International Ltd., Rotkreuz, Switzerland) and collagenase (1 mg/ml; Worthington, Lakewood, NJ) in RPMI 1640 for 60 min at 37°C. The suspension was then filtered through a cell strainer, and, subsequently, washed by centrifugation in PBS to remove residual enzyme. To obtain mononuclear cells (MNCs), plaque cell suspensions or blood underwent Ficoll-Hypaque (Sigma-Aldrich, St. Louis, Missouri) density-gradient centrifugation.

### Flow Cytometry

The following mouse anti-human mAbs were used in our study: anti-CD3 PerCp Cy5.5 FITC (clone UCHT1), -CD16 PE-Cy7 (clone 3G8), -CD56 APC (clone NCAM 16.2), -CD94 FITC (clone HP-3D9), -HLA-DR FITC/APC-H7/BV421 (clone G46-6), -CD11c PerCP-Cy5.5 (clone B-ly6), -CD45 APC-H7 (clone 2D1), -Perforin FITC (clone δG9), -CD103 BV421(clone Ber-ACT8), -CD49a PE (clone SR84), -CD69 APC/APC-H7 (clone FN50), -CD57 PE (clone NK-1), -CD19 FITC (clone HIB19) from BD Biosciences (San Jose, CA); -perforin FITC (clone delta G9) from Ancell (Stillwater, MN); -CD3 VioGreen (clone REA 613), -NKG2A PE-Vio770 (clone REA 110), -NKG2C VioBright-FITC (clone REA205), -CD11c (clone MJ4-27G12), and –MICA/B PE-Vio770 (clone 6D4), -BDCA-2 FITC (clone AC144), -NKp30-PE (clone AF29-4D12) from MiltenyiBiotec (Bergisch Gladbach, Germany); -ULBP-3 PE (clone 166510), -B7-H6 APC (clone 875001) from R&D (Minneapolis, MN); -CD16 Pacific Blue (clone 3G8) from Beckman Coulter (Brea, CA). After incubation with the relevant mAbs for 20 min at 4°C, cells were then washed and analyzed by flow cytometry. Negative controls included isotype-matched irrelevant Abs. Intracellular staining with anti-human perforin-FITC was performed using the Fix/Perm kit from BD Biosciences (San Jose, CA) according to the manufacturer's indications. Samples were then acquired using FACSCantoII (BD Biosciences, San Jose, CA) and analyzed by FlowJo 9.0.2 software (Tree Star).

### Cytokine Production Assay

For the analysis of cytokine production, NK cells from carotid plaques or autologous PB were stimulated with 10 ng/mL phorbolmyristate acetate (PMA) and 500 ng/mL ionomycin (both from Sigma-Aldrich, St. Louis, Missouri). After 45 min, Golgi Stop and Golgi Plug (both from BD Bioscience, San Jose, CA) were added and stimulation was allowed to continue for a total of 4 h. At the end of the stimulation, cells were first stained for relevant surface markers and finally fixed and permeabilized for intracellular detection of IFN-γ expression by specific mAb (BD Biosciences, San Josè, CA).

### RT-PCR Analysis

Total RNA was extracted from carotid plaques. Briefly, samples were homogenized with Tissue Ruptor (Qiagen, Hilden, Germany) and subsequently processed using RNeasy Fibrous Tissue Mini Kit (Qiagen, Hilden, Germany). cDNA was prepared using the Real Master Script Super Mix Kit (5-PRIME) following manufacturer's instructions. Amplifications were performed for 30 or 35 cycles utilizing Platinum TAQ DNA Polymerase (ThermoFisher, Waltham, Massachusetts) with an annealing T of 58°C. Primers used were: β-actin for 5′ ACTCCATCATGAAGTGTGACG and β-actin rev 5′ CATACTCCTGCTTGCTGATCC; MICA for 5′TACGATGGGGAGCTCTTC and MICA rev 5′ GACCCTCTGCAGCTGATG; MICB for 5′ TCCCGGCATTTCTACTAC and MICB rev 5′ TGCATCCCTGTGGTCTCC; H6 for 5′ TGCTGTGGGCGCTGACGA and H6 rev 5′ GGTAGAACCCACTTGACTCA; ULBP-1 for 5′ TGCAGGCCAGGATGTCTTGT and ULBP-1 rev 5′ CATCCCTGTTCTTCTCCCACTTC; ULBP-2 for 5′ CCCTGGGGAAGAAACTAAATGTC and ULBP-2 rev 5′ ACTGAACTGCCAAGATCCACTGCT; ULBP-3 for 5′ ATTCTTCCGTACCTGCTATT and ULBP-3 rev 5′ GCTATCCTTCTCCCACTTCT. PCR products (249 bp fragment for β-actin, 618 bp for MICA, 698 bp for MICB, 462 bp for B7-H6, 171 bp for ULBP-1, 199 bp for ULBP-2, and 492 bp for ULBP-3) were separated by electrophoresis on a 1.5% (w/v) agarose gel and visualized by ethidium bromide staining.

### ELISA

Sera were processed using a serum separator tube (SST) and samples were allowed to clot for 2 h at room temperature before centrifugation for 15 min at 1,000 × g. Samples were stored at −20°C prior analysis. To analyze the soluble ligands of NK activating receptor MICA, ULBP-3 and B7-H6 (sMICA, sULBP-3, and sB7-H6), ELISA was performed following manufacturer's instructions using the following reagents: ULBP-3 ELISA KIT (Cusabio, Hubei Province, CN), MICA Duo ELISA Set (R&D, Minneapolis, MN); B7-H6 Elisa Kit (MyBioSource, Inc., San Diego, CA).

### Statistical Analysis

We applied Kruskal-Wallis tests for evaluating quantitative variables adjusting for multiple comparison using Dunn's correction. Graphic representation and statistical analysis were performed using GraphPad Prism 6 (GraphPad Software La Jolla, CA). Pearson correlation index was used to calculate correlation for pair-wise continuous variables.

## Results

### Clinical Characteristics of Patients

Out of 50 patients 16 were women (32%). Mean age was 71 ± 10 years (range 46–85). Twenty-one patients (42%) were diabetic, 28 (56%) were smokers, 35 (70%) had hypertension, and 31 (62%) had hyperlipidemia. Thirty-one patients were asymptomatic (62%) and 19 (38%) were symptomatic. Clinical characteristics of patients and control individuals are summarized in Table 1.

**Table 1:** Clinical characteristics of atherosclerotic patients and controls.

	**Symptomatic (*n* = 19)**	**Asymptomatic (*n* = 31)**	**Controls (*n* = 15)**
Age	72.6y (4.4)	71.4y (5.8)	69.0y (6.7)
Women	5 (26.3%)	11 (35.4%)	5 (33%)
Stroke	15 (78.9%)	0	0
TIA	3 (15.7%)	0	0
Amaurosis fugax	1 (5.2%)	0	0
Smoker	8 (42.1%)	20 (64.5%)	7 (47%)
Hypertension	15 (78.9%)	20 (64.5%)	8 (54%)
Diabetes status	7 (36.8%)	14 (45.1%)	5 (33%)
Hyperlipidemia	16 (84.2%)	15 (48.3%)	7 (47%)
Ischemic: heart disease	11 (57.8%)	8 (25.8%)	0
Plaque stenosis			
70–80%	2 (10.5%)	3 (9.6%)	0
>80%	17 (89.4)	28 (90.3)	0

*Categorical variables were reported as count (percentage). Continuous variable was reported as mean ± standard deviation*.

### Symptomatic Carotid Plaques Are Infiltrated by a Higher Number of NK Cells

**Figure 1:**
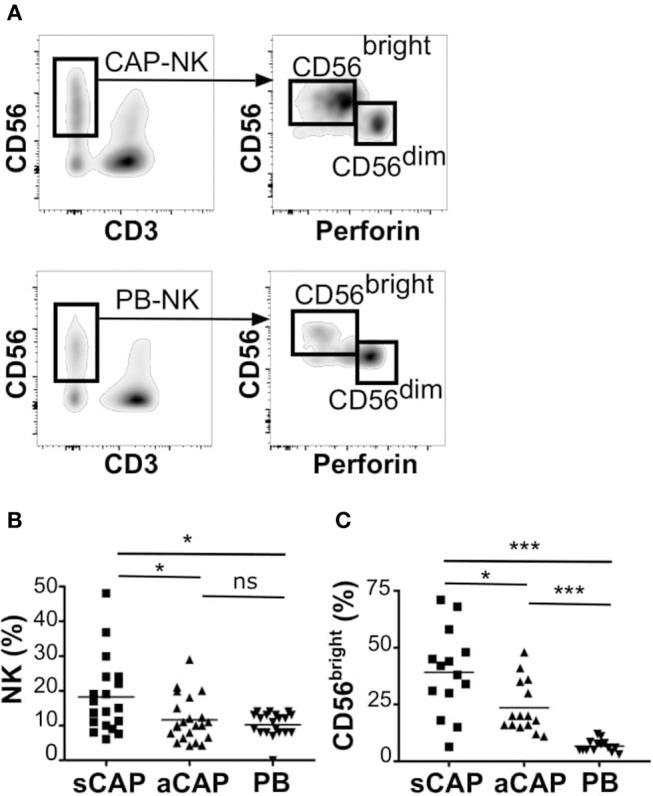
Characterization of NK cells from carotid atherosclerotic plaques (CAP-NK). After processing carotid plaques, atheroma-resident mononuclear cells were stained with relevant mAbs and analyzed by flow cytometry gating on CD45^+^ lymphocytes. **(A)** Comparative analysis of total NK cells and of NK cell subsets (i.e., CD56^bright^perforin^low^ and CD56^dim^perforin^high^ cells) from carotid atherosclerotic plaques (CAP-NK) and autologous PB (PB-NK). **(B)** Percentage of total NK cells present in carotid plaques gating on CD45^+^ lymphocytes from symptomatic (sCAP-NK) and asymptomatic (aCAP-NK) patients. **(C)** Percentage of CD56^bright^ NK cells present in carotid plaques gating on total NK cells. In **(B)** and in **(C)** NK cells from autologous peripheral blood (PB-NK) were also analyzed as control. Kruskal-Wallis test was used to determine statistical significance for group comparison. n.s., non-significant; ^*^p < 0.05 and ^***^p < 0.001.

**Figure 2:**
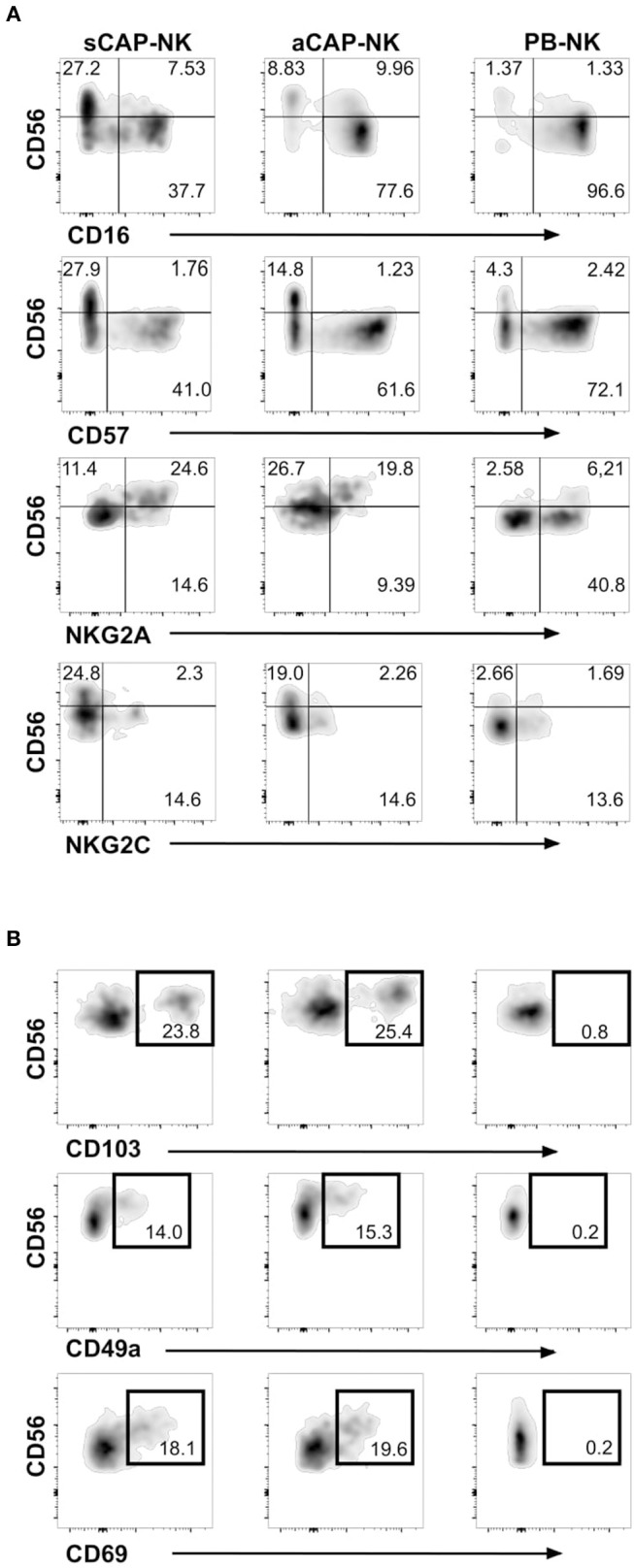
Expression of NK cell receptors and tissue resident markers on NK cells from carotid atherosclerotic plaques (CAP-NK). **(A)** Expression of different NK cell surface molecules was analyzed on carotid plaque NK cells from symptomatic patients (sCAP-NK), asymptomatic patients (aCAP-NK), and on peripheral blood NK cells (PB-NK) of the same patients by flow cytometry. Data shown are representative of at least five carotid plaques showing similar results. No main differences were observed in PB of symptomatic and asymptomatic patients. **(B)** Expression of tissue resident markers (i.e., CD103, CD49a, and CD69) was analyzed on NK cells from sCAP-NK, aCAP-NK, and PB-NK. Data shown are representative of at least five carotid plaques showing similar results.

To evaluate the presence of NK cells, we performed flow cytometric analysis of cell suspensions obtained upon dissociation of CAP from both symptomatic and asymptomatic patients. NK cells isolated from CAP of 41 patients affected by atherosclerotic disease of carotid artery (19 symptomatic and 22 asymptomatic) were analyzed in comparison to NK cells from autologous PB. Since NK cells share their CD56 expression with group 3 innate lymphoid cells (ILC3) (30), this latter subset was excluded from the analyses on the basis of the absence of CD94 and perforin (15, 30, 31). NK cells were consistently detected in all the examined samples and we assessed the relative frequency of the two main NK cell subsets, namely CD56^bright^perforin^low^ and CD56^dim^perforin^high^ NK cells. Due to the possible downregulation of CD16 on NK cells extracted from solid tissues (32, 33), we distinguished CD56^dim^ from CD56^bright^ NK cells on the basis of perforin expression (Figure 1A). NK cell frequency was higher in CAP isolated from symptomatic patients whereas CAP from asymptomatic patients showed NK cell frequency similar to PB (Figure 1B). NK cells isolated from CAP of both symptomatic (sCAP) and asymptomatic (aCAP) patients displayed a higher frequency of CD56^bright^perforin^low^ NK cells when compared to autologous PB, and noteworthy, sCAP showed the highest frequency of CD56^bright^ perforin^low^ NK cells (Figure 1C). As previously reported (16), no significant difference in the frequency of NK cell subsets was observed between PB of atherosclerotic patients and controls. In line with their constitutive low expression of intracellular perforin, CD56^bright^ NK cells also presented low or undetectable surface expression of CD16 and CD57, while they were typically positive for NKG2A (Figure 2). Both NKG2D and NKp30 activating receptors were homogenously expressed in all the samples analyzed. Within the CD56^dim^ subset a consistent fraction of NK cells expressed NKG2C (Figure 2A and Figure S1A), while markers of lymphocyte residency, such as CD103 (also known as αE integrin), CD49a (also known as α1 integrin), and CD69 (14), appeared confined to the CD56^bright^ NK cell subset (Figure 2B), suggesting a preferential migration of CD56^bright^ NK cells into CAP. However, no significant differences were observed for these molecules between symptomatic and asymptomatic patients (Figure S1B).

### Carotid Plaque Tissues Express Ligands for NK Cell Activating Receptors

**Figure 3:**
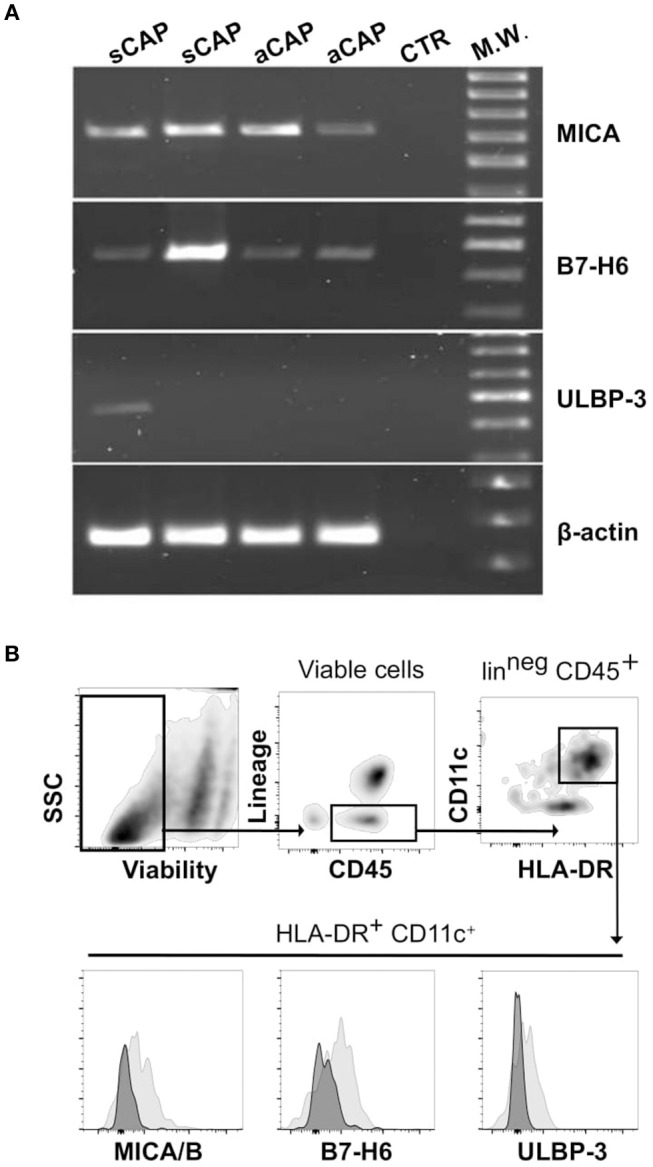
Expression of cellular ligands for NK cell activating receptors within carotid atherosclerotic plaques (CAP). **(A)** mRNA expression of MICA, B7-H6, and ULBP-3 was analyzed in CAP-specimens by PCR. Primers specific for β-actin were utilized as positive control. Data shown are from atheroma specimens obtained from four different patients, two symptomatic (sCAP) and two asymptomatic (aCAP), and are representative of results obtained with 10 different patients. **(B)** After processing CAP to single cell suspension, mononuclear cells were analyzed by flow cytometry for the expression of the main NK activating receptor ligands, which were detected on a CD45^+^HLA-DR^+^CD11c^+^lin (CD3, CD19, CD94)^neg^ cell population (gray histograms). Negative controls included isotype-matched irrelevant mAbs (dark histograms).

Having observed the presence of distinct populations of NK cells in CAP, a main question remained whether ligands for their activating receptors were expressed by CAP tissues. In agreement with previous reports (19), we found CAP tissues consistently expressed significant levels of mRNA for MICA, a main ligand for NKG2D receptor. In addition, we found that B7-H6, a main cellular ligand for NKp30 receptor (34), was also expressed in carotid plaques (Figure 3A). MICB and ULBPs mRNA were detectable only in a fraction of carotid plaques analyzed, and, among ULBP molecules, ULBP-3 was the most frequently observed (Figure 3A and Figure S2).

Thus, considering these results, we processed CAP tissues and analyzed by flow cytometry, on the isolated cells, the protein expression of the most represented ligands, revealing that MICA/B (6 pts out of 10), ULBP-3 (4 pts out of 10), and B7-H6 (6 pts out of 10) were detectable in a CD45^+^HLA-DR^+^CD11c^+^lin (CD3, CD19, CD94)^neg^ cell population resident within CAP (Figure 3B).

It is worth to note that although mRNA expression of these ligands was detectable in the majority of the samples, protein expression was detected in a smaller fraction, which might reflect technical impediments associated to CAP tissue processing.

### Sera of Symptomatic CAP Patients Contain Higher Levels of Soluble Ligands for Activating NK Cell Receptors

Metalloproteases (MPPs) have been reported to play a role in inducing AP instability (35, 36). Because these enzymes can also cause the shedding of cellular ligands for NK activating receptors (37–39), we investigated whether symptomatic CAP could be associated with the release of soluble forms of NK activating receptor ligands detected in CAP.

**Figure 4:**
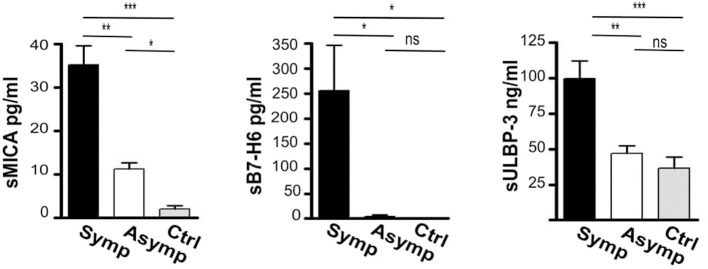
Assessment of soluble ligands for NK cell activating receptors in the sera of patients affected by carotid atherosclerotic plaques. Soluble ligands for NK activating receptors (sMICA, sB7-H6, sULBP-3) were assessed in the serum of symptomatic (Symp, black bars), of asymptomatic (Asymp, white bars) patients and of controls (Ctrl, gray bars) by ELISA; Bars represent mean values ± SD from 10 symptomatic, 19 asymptomatic patients and 10 control individuals. Kruskal-Wallis text was the used to determine statistical significance for group comparison. n.s., non-significant; ^*^p < 0.05, ^**^p < 0.01, and ^***^p < 0.001.

Soluble forms of the ligands were consistently increased in the serum of atherosclerotic patients and sera levels of both sMICA and sULBP-3 were significantly higher in symptomatic patients (fold of increase CAP/Controls: 10 and 5.7 for sMICA and sULBP-3, respectively) (Figure 4). In addition, symptomatic patients displayed significant levels of sB7-H6, which were undetectable in both asymptomatic patients and controls (Figure 4).

### IFN-γ Production by CAP-NK Is Associated With Serum Levels of Soluble MICA

**Figure 5:**
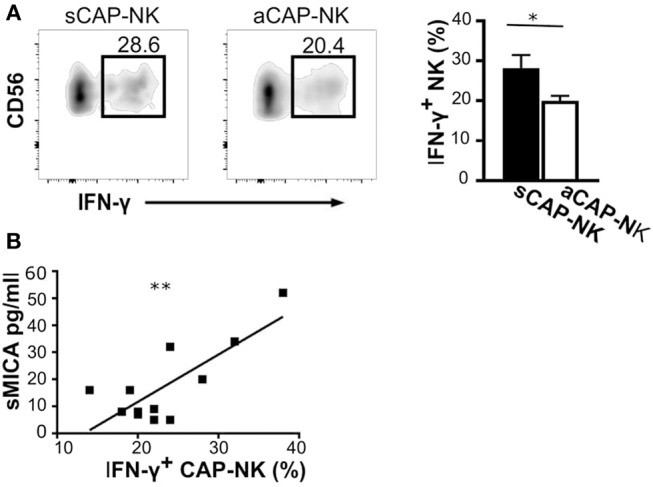
IFN-γ production by NK cells isolated from carotid plaques (CAP-NK). **(A)** IFN-γ production was assessed in NK cells from carotid plaques of symptomatic (sCAP-NK) and asymptomatic (aCAP-NK) patients after 4 h of stimulation with PMA and ionomycin. Dot plots shown are representative of 12 patients (five symptomatic and seven asymptomatic). Kruskal-Wallis test was used to determine statistical significance for group comparison. Bars represent mean value ± SD of the frequency of IFN-γ^+^ NK cells in carotid plaques from symptomatic (white bar) and asymptomatic patients (black bar). **(B)** The Pearson correlation coefficient was calculated between serum levels of soluble MICA and IFN-γ^+^ NK cells isolated from CAP. ^*^p < 0.05 and ^**^p < 0.01.

MMPs have been reported to be induced by IFN-γ (40), a major cytokine produced by activated NK cells. Having observed that CAP as well as sera of atherosclerotic patients contained the ligands for NK activating receptors, we investigated whether IFN-γ production by CAP-NK cells might be associated with either plaque stability or release of soluble NK activating ligands. We observed that NK cells isolated from sCAP were more prone to produce IFN-γ (Figure 5A). Remarkably, the frequency of IFN-γ^+^ NK cells in CAP positively correlated with serum levels of sMICA (Figure 5B), although no significant correlation was observed for other soluble ligands.

## Discussion

Although previous studies demonstrated a functional role of NK cells in different experimental models of atherosclerosis, recent data derived from hypercolesterolemic mice failed to find a definite role for NK cells in atherosclerosis development. Nevertheless, they also demonstrated that NK cells might exacerbate atherosclerosis in case of activation induced by viral infections (17), an event frequently occurring throughout the whole life course. In agreement with this previous report, chronic viral infections by CMV, which strongly activate NK cells, have been shown to contribute to the inflammatory process leading to the formation of AP (41–43) as well as to increase the risk of vascular complications. Thus, it is conceivable that activation of NK cells in these inflammatory contexts might contribute to exacerbate atherosclerosis.

Because most of the current knowledge on human NK cells in AP has been derived from investigations of atherosclerotic lesions by immunohistochemistry, information on phenotypic and functional features of NK cells involved in this pathology was so far limited. Given the substantial functional differences between the two main human circulating NK cell subsets, the lack of information regarding the relative distribution of CD56^bright^ and CD56^dim^ NK cells has so far represented a major limitation for a comprehensive understanding of the functional role of NK cells within the immune network ruling CAP development. In this study, we identified NK cells in CAP by multiparametric flow cytometric analysis, also corroborating previous results obtained by IHC (1, 44, 45).

The analysis of NK cell subsets revealed that CAP were enriched in CD56^bright^ perforin^low/neg^ NK cells as compared with autologous PB. The frequency of CD56^bright^ NK cells was even higher in plaques of symptomatic patients compared to that of asymptomatic ones, suggesting their preferential accumulation in the microenvironment of unstable plaques or, on the other hand, their potential contribution in plaque destabilization. This finding is in line with the distribution of chemokine receptor ligands leading to NK cell migration in AP, as it has been recently demonstrated that CCL19 and CCL21, the major chemokines dictating CCR7-dependent migration of CD56^bright^ NK cells, are upregulated in atherosclerotic carotid plaques of symptomatic patients as compared to asymptomatic ones (46, 47). Remarkably, we observed that CD56^bright^ CAP-NK cells selectively express variable levels of tissue resident markers, i.e., CD103, CD49a, CD69, which are absent in the CD56^dim^subset as well as in PB-NK cells, suggesting that CD56^bright^ NK cells might preferentially be recruited to the plaque and then up-regulate markers of tissue residency under the influence of local inflammation. Our current observations are also in agreement with former studies showing that CD56^bright^ NK cells, a cell subset particularly prone to cytokine production, often represent the main NK cell population in other inflamed tissues (48).

In line with previous reports showing the expression of MICA/B in atherosclerotic lesion (19) and of B7-H6 under inflammatory conditions (21), we observed that MICA/B, B7-H6, and ULBP-3 were expressed in CAP by large/scattered cells of myeloid origin compatible with a macrophagic/dendritic cell phenotype. It is likely that foam cells, derived from monocytes recruited upon inflammatory conditions within the intima of atherosclerotic lesions, might express ligands for activating NK receptors, since this event has been shown to occur, at least for some NK activating receptor ligands, in the presence of oxidized low density lipoproteins (20, 21). However, we could not exclude that other cell subsets residing within carotid plaques or endothelial cells might also express these ligands in the inflammatory microenvironment of the vessel. Further analyses are needed to explore in deeper details AP cellular components that might represent cell targets for NK cells.

Overall, our observations might suggest the existence, within the atherosclerotic lesions, of an inflammatory pathway sustained by NK cells recognizing their activating ligands on macrophage-like cells or other cells expressing the ligands in CAP tissue, with a consequent release of IFN-γ able to boost the inflammatory process. This assumption is also supported by previous reports describing in AP direct contacts between CD56^+^ cells, assumed to be *bona fide* NK cells, and macrophages (1). Thus, because CAP-NK cells constitutively express both NKp30 and NKG2D at levels similar to PB-NK (Figure S3), they can be actively triggered by target cells expressing ligands for these receptors within CAP.

Remarkably, NK cells in symptomatic patients were stronger IFN-γ producers. Given that IFN-γ has been shown to behave as a pro-atherogenic cytokine, also causing plaque destabilization through either the induction of smooth muscle cell apoptosis or the release of MMPs (40), it might be envisaged that CAP-resident NK cells might contribute to both plaque formation and destabilization by massively secreting this cytokine. IFN-γ produced by CAP-NK cells, by inducing MMP production, might also contribute to determine ligand shedding. This hypothesis is further sustained by the observation that the frequency of IFN-γ^+^ NK cells correlated with serum levels of sMICA.

In conclusions, our data indicate that CD56^bright^ NK cells might play a role in the progression of the atherosclerotic process within carotid plaques by exerting a pro-inflammatory effect upon recognition of cell targets within CAP. The evidence that soluble forms of NK activating receptor ligands are both increased in symptomatic patients and associated with the amount of CAP IFN-γ^+^ NK cells, also favors the hypothesis that NK cell-derived IFN-γ might contribute to the production, within AP, of MMPs able, on the one hand, to shed these soluble forms (38, 39) and, on the other, to critically affect CAP stability (35, 36) and hence its clinical consequences.

## Data Availability

All datasets generated for this study are included in the manuscript and/or the Supplementary Files.

## Ethics Statement

This study was approved by local Ethical Committee (Comitato Etico di Messina prot. 59/18).

## Author Contributions

IB planned and performed most experiments, analyzed data, and wrote the manuscript. DS, CB, NP, and FB provided critical biological samples and patient data. CC established new methods, performed experiment, analyzed the data, and revised the manuscript. CD and DO collected human samples and performed experiments. RC provided unique reagents and advised on their use. DS, PC, and FB supervised the project, analyzed data, and revised the manuscript. GF planned experiments, supervised the project, analyzed data, and wrote the manuscript.

### Conflict of Interest Statement

The authors declare that the research was conducted in the absence of any commercial or financial relationships that could be construed as a potential conflict of interest.

## Abbreviations

KIR: killer Ig-like receptors
MMPs: metalloproteases
NCRs: natural cytotoxicity receptors
NK: natural killer
AP: atherosclerotic plaques
CAP: carotid atherosclerotic plaques
MMP: metalloprotease
IHC: immunohystochemistry
IFN-γ: interferon-gamma.
