# Supplementary figures and images for: Symptomatic Carotid Atherosclerotic Plaques Are Associated With Increased Infiltration of Natural Killer (NK) Cells and Higher Serum Levels of NK Activating Receptor Ligands
Source: Front Immunol. 2019 Jul 12;10:1503. doi: 10.3389/fimmu.2019.01503 (PMC6639781; doi:10.3389/fimmu.2019.01503)

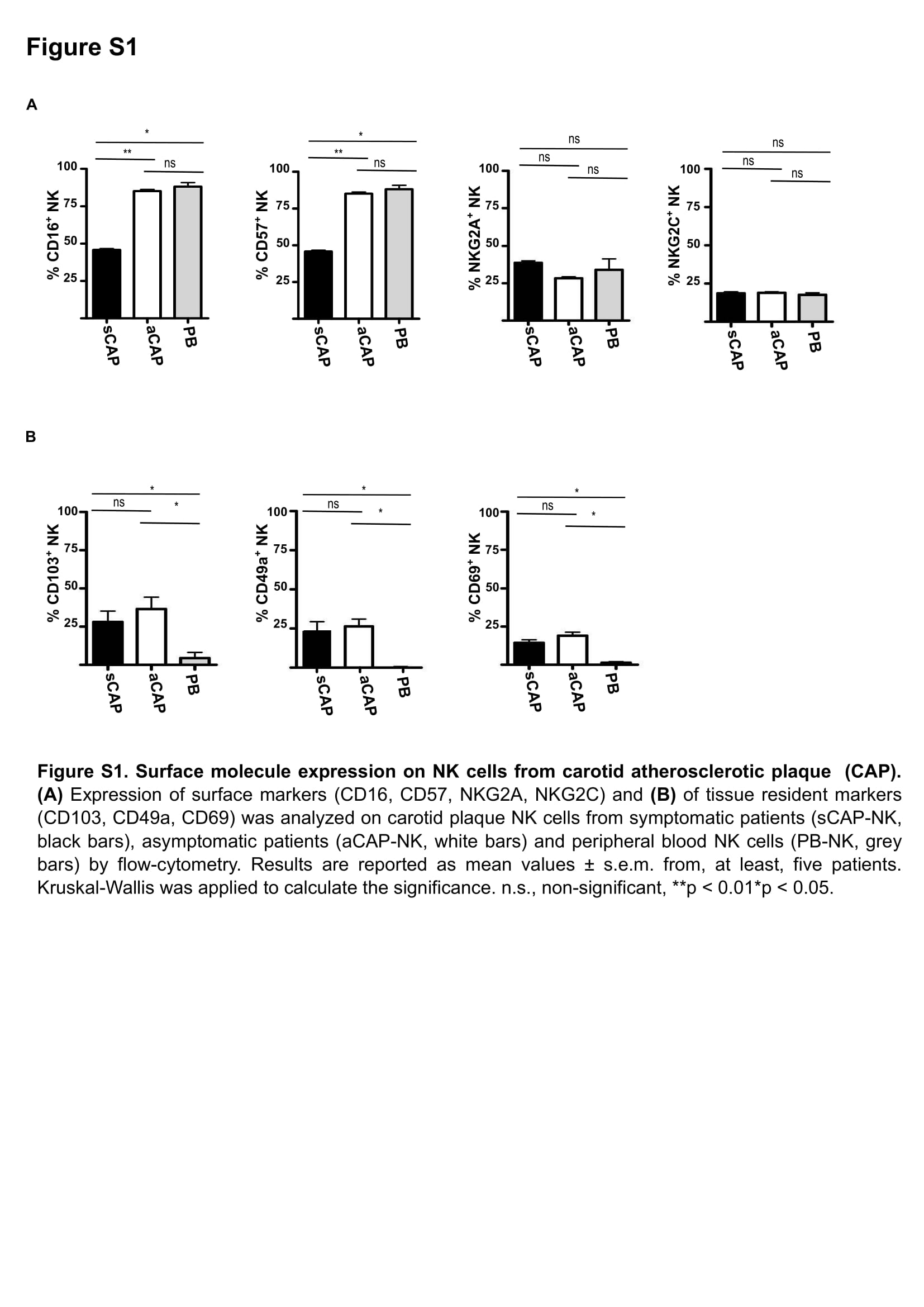

Supplement: Supplementary file 1 [file Image_1.JPEG]

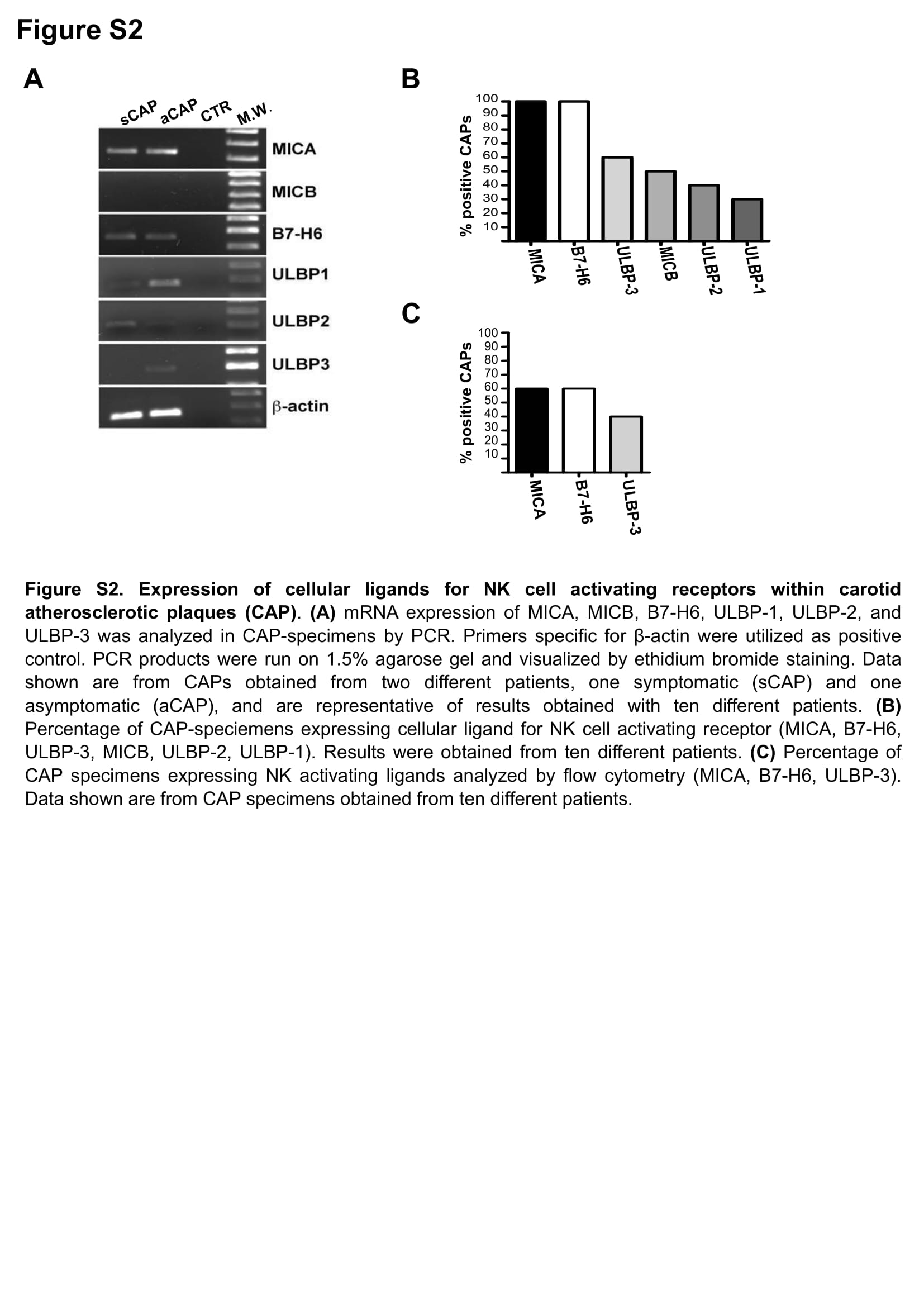

Supplement: Supplementary file 2 [file Image_2.JPEG]

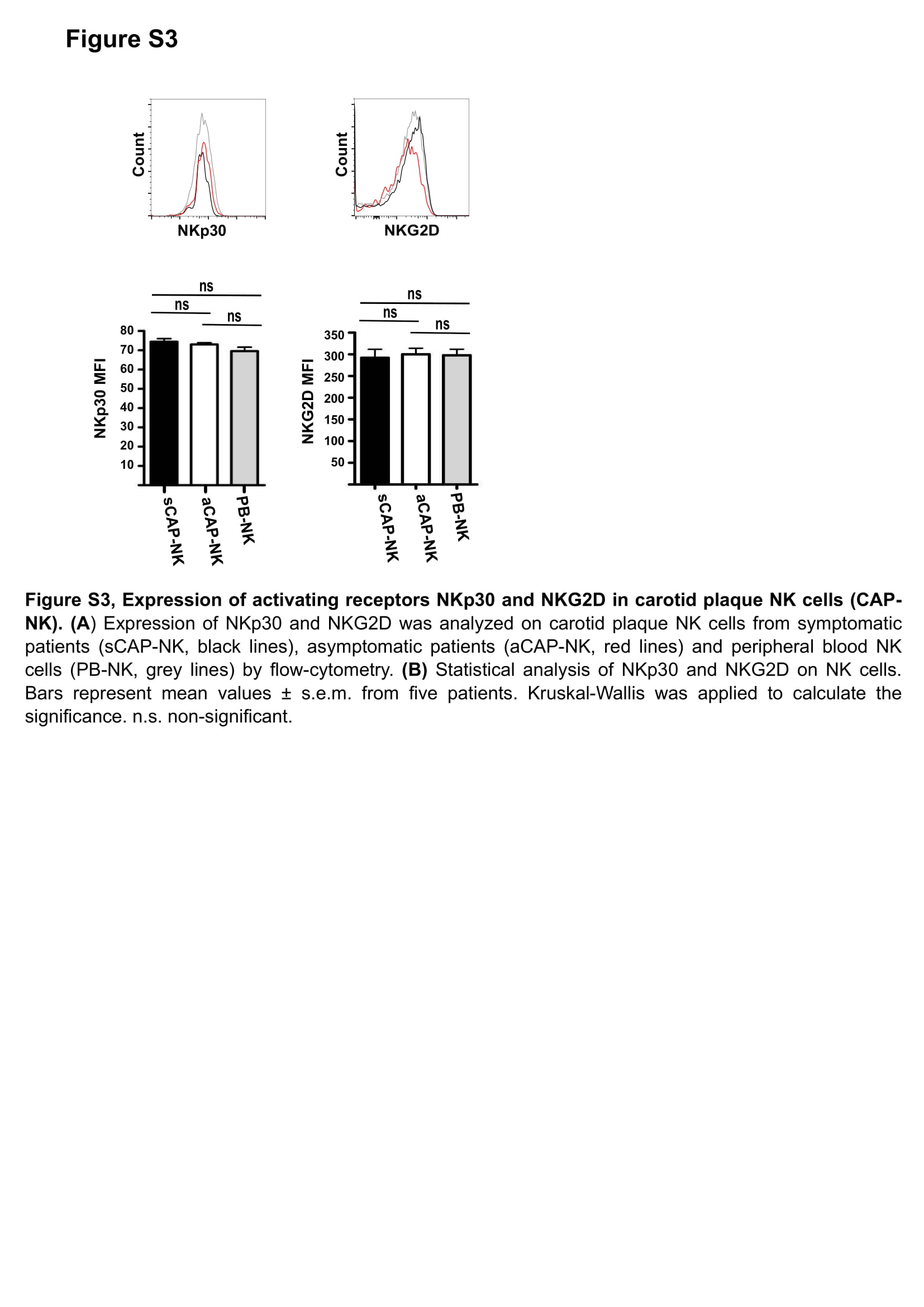

Supplement: Supplementary file 3 [file Image_3.JPEG]
